# Genome and epigenome editing identify *CCR9* and *SLC6A20* as target genes at the 3p21.31 locus associated with severe COVID-19

**DOI:** 10.1038/s41392-021-00519-1

**Published:** 2021-02-22

**Authors:** Yao Yao, Fei Ye, Kailong Li, Peng Xu, Wenjie Tan, Quansheng Feng, Shuquan Rao

**Affiliations:** 1 School of Basic Medicine, Chengdu University of Traditional Chinese Medicine, Chengdu, China; 2 Division of Hematology/Oncology, Boston Children’s Hospital, Department of Pediatric Oncology, Dana-Farber Cancer Institute, Harvard Medical School, Boston, MA USA; 3 NHC Key Laboratory of Biosafety, National Institute for Viral Disease Control & Prevention, Chinese Center for Disease Control and Prevention, China CDC, Beijing, China; 4 Children’s Medical Center Research Institute, University of Texas Southwestern Medical Center, Dallas, TX USA; 5 Hematology Center of Cyrus Tang Medical Institute, Soochow University, Suzhou, China

**Keywords:** Respiratory tract diseases, Functional genomics

**Dear Editor,**

The coronavirus disease 2019 (COVID-19) pandemic caused by severe acute respiratory syndrome coronavirus 2 (SARS-CoV-2) is a major global health threat. Mortality is predominantly caused by severe respiratory failure related to interstitial pneumonia in both lungs and acute respiratory distress syndrome. Recently, genome-wide association studies (GWASs) have identified chromosome 3p21.31 (sentinel variant: rs11385942) to be associated with both severe and general affected COVID-19 cases.^1^ Despite of several coding genes mapped to the chromosome 3p21.31 locus (Fig. 1a), the causal genes responsible for this locus remains largely illusive. Here, we performed both genome and epigenome editing approaches in potentially disease-relevant cell types to identify the causal genes regulated by this locus.

**Fig. 1:**
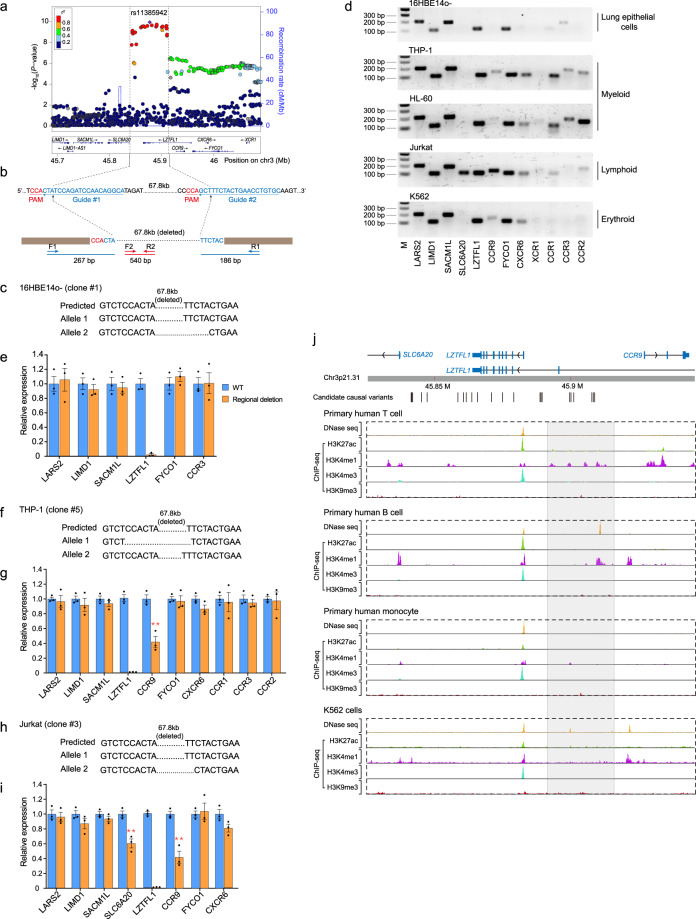
CRISPR/Cas genome editing identified *CCR9* and *SCL6A20* as target genes responsible for the chromosome 3p21.31 locus associated with severe COVID-19. **a** Regional association plots of the chromosome 3p21.31 locus associated with severe COVID-19 with respiratory failure. Association data was from Ellinghaus et al. study^1^. The linkage disequilibrium values were calculated on the basis of the 1000 Genomes project (European panel). The positions in the genome assembly hg19 are plotted. The recombination rate is shown in centimorgans (cM) per million base pairs (Mb). The purple diamond represents the variant most strongly associated with severe COVID-19 and respiratory failure. **b** Schematic diagram of genome editing strategy. The positions of sgRNAs and PAM sequences are shown in blue and red, respectively. The locations of PCR primers are indicated by arrows. F, forward; R, reverse. Clones of bi-allelic deletion are indicated by the presence of PCR product from F1+R1, but absence of PCR product from F2+R2. **c**, **f**, **h** Sequence of isogenic clonal deletions of the chromosome 3p21.31 locus obtained using CRISPR/Cas9 genome editing in 16HBE14o-, THP-1, and Jurkat cells, respectively. “Predicted” sequence indicates 67.8-kb deletion without any indel mutagenesis. For clones with bi-allelic deletion (see Fig. S3), genomic sequences were confirmed by cloning of each allele followed by Sanger sequencing. **d** Genes within 1 megabase covering the chromosome 3p21.31 locus, expressed in different cell types, were ascertained by RT-PCR. **e**, **g**, **i** Altered gene expression in clonal isogenic deletions in 16HBE14o-, THP-1, and Jurkat cells, respectively. RT-qPCR analysis of genes within 1 megabase covering the chromosome 3p21.31 locus was performed. Student’s *t* test was performed for each gene (*n* = 3 technical replicates). *P < 0.05, **P < 0.01. **j** Epigenetic programming at the chromosome 3p21.31 locus. Density maps are shown for DNase-Seq, ChIP-seq of active histone marks (H3K27ac and H3K4me1), repressive H3K9me3, and promoter mark (H3K4me3), in primary human T cells, B cells, and monocytes, as well as K562 cell lines. Locations of candidate causal variants from fine-mapping analysis were indicated by short vertical lines. Genomic locations in hg19 presented. Epigenetic datasets were obtained from the ENCODE Project (see Supplementary methods)

Given that lung injury is one of the main clinical characteristics of patients with severe COVID-19, we first chose human bronchial epithelium cells (16HBE14o-) to investigate the regulatory effect of GWAS signals on target genes at chromosome 3p21.31. A total of 22 candidate causal variants for the 3p21.31 locus were prioritized through Bayesian fine-mapping analysis,^1^ making it infeasible to interfere with these causal variants individually. We instead utilized CRISPR/Cas9 with flanking single-guide RNAs (sgRNAs) to delete a 67.8-kb genomic region covering all candidate causal variants, as illustrated in Fig. 1a. Clones with bi-allelic genomic deletion (Δ67.8 kb) were confirmed by both PCR and Sanger sequencing (Fig. 1c, Supplementary Fig. 1 and Supplementary Table 1), and expression of 12 genes within 1 Mb surrounding rs11385942 were analyzed by RT-qPCR. No differential gene expression was observed between Δ67.8-kb and WT clones, except *LZTFL1* (Fig. 1c and e). As shown in Supplementary Fig. 2a, three SNPs near the *LZTFL1* gene locus, including rs17713054, rs13078854, and rs71325088, were observed overlying chromatin accessibility region and histone modifications with an enhancer signature, including the presence of H3K4me1 and H3K27ac and absence of H3K4me3 marks, in human lung epithelial cells annotated in the ENCODE project.^2^ To understand whether variants at the 3p21.31 locus have regulatory effect on *LZTFL1* gene expression, we employed CRISPRi to deactivate the potential enhancer downstream the *LZTFL1* gene locus, which, however, did not result in significant change of *LZTFL1* expression (Supplementary Fig. 2b–d). Together, these data suggested that *LZTFL1* may not be the target gene of the 3p21.31 locus, and human lung epithelial cells may not be the driver of COVID-19 pathogenesis, but instead the consequence of the disease pathogenic process.

Previous studies have suggested that dysregulated innate immune response with exaggerated inflammatory cytokine production is a defining driving severe COVID-19 disease.^3^ Upon SARS-CoV-2 infection, there is an accumulation of immune cells in the lungs in severe COVID-19 patients, especially monocyte, monocyte-derived macrophages, neutrophils, and proliferating T cells.^3^ We therefore focused further experiments to characterize regulatory effects of the 3p21.31 locus in immune cells. Four different cell lines, THP-1, HL-60, Jurkat, and K562, which can closely resemble human monocytes, promyelocyte (granulocyte precursor), T lymphocytes, and erythroid, were utilized in the present study due to their ease of genomic manipulation. As shown in Fig. 1c, detectable gene expression was observed for all genes, except *XCR1*, within 1 Mb surrounding the 3p21.31 locus, in either myeloid or T lymphoid cells. Again, CRISPR/Cas9 genome editing with sgRNA pair flanking the peak association signal at 3p21.31 was employed to create bi-allelic clonal deletions (Δ67.8 kb) in both THP-1 and Jurkat cells (Fig. 1f, h and Supplementary Fig. 3). In each cell line, we investigated the expression of all genes within 1 Mb. Compared with WT clone, loss of 67.8 kb resulted in consistent suppression of *CCR9* gene expression (*P* < 0.01) in both THP-1 and Jurkat cells (Fig. 1g, i). Despite exclusive expression in Jurkat cells, the *SLC6A20* gene was also downregulated in Δ67.8 kb compared with WT clones. In order to better account for heterogeneity resulting from expansion of single-cell clones, another Jurkat cell clone with double allele deletion was chosen as cross controls. We determined that expression of both *SLC25A6* and *CCR9* were affected in Δ67.8 kb compared with WT clones, although *CCR9* showed the largest consistent suppression (Supplementary Fig. 3c). These data strongly suggested that both *CCR9* and *SLC6A20* are potential target genes responsible for the 3p21.31 locus.

To further understand the regulatory effect of the 3p21.31 locus on *CCR9* and *SLC6A20*, we next verified the presence of active chromatin states overlapping the fine-mapped candidate causal variants in primary immune cells and K562 cell lines. We showed that six of the fine-mapped variants (rs34326463, rs76374459, rs73064425, rs13081482, rs35652899, and rs35044562) overlaps human T-cell specific primed enhancers marked by H3K4me1 peak and depletion of H3K4me3 (Fig. 1j). The genotypes of these variants, as well as other candidate causal variants at the 3p21.31 locus in Jurkat cells, are shown in Supplementary Fig. 4. Since T cells may utilize highly dynamic enhancer repertoire in response to infections, with a substantial portion of enhancers decommissioned and a large number of new enhancers activated during T cells responses, it’s highly plausible that this enhancer would be activated during SARS-CoV-2 infection, and variants within this enhancer could result in differential gene expression of *CCR9* and *SLC6A20*, thus mediating variable severity of COVID-19. SLC6A20 can functionally interacts with angiotensin-converting enzyme 2, the SARS-CoV-2 cell-surface receptor.^4^ CCR9 has been described as a homeostatic and inflammatory chemokine receptor. Expressed by T lymphocytes and eosinophils, CCR9 plays a key role in regulating cell recruitment and modulating inflammatory process during pathogen infection.^5^ Supporting our results, significantly decreased expression of CCR9 was observed in COVID-19 patients, compared with healthy controls, in peripheral blood mononuclear cells (*P* = 8.74E−05; Supplementary Fig. 5).^5^

Collectively, by utilizing CRISPR/Cas9 mediated genomic deletion, we identified *CCR9* and *SLC6A20* as potential target genes of the 3p21.31 locus. Epigenomic data from human primary immune cells revealed an enhancer signature overlapping six candidate causal variants exclusively in T lymphocytes across a cohort of investigated immune cells, suggesting a regulatory function for these variants in T lymphocytes. Notably, due to limited epigenetic datasets, we cannot exclude the possibility of regulatory functions of the 3p21.31 locus in other cell types. Moreover, unexpected chromatin structure disruption, i.e., gene conversion and segmental deletion, due to long genomic fragment deletion, would probably affect the transcription of downstream genes. Last but most important, despite the target genes were identified through genomic deletion, the regulatory mechanisms underlying the causal variants at the 3p21.31 locus remains illusive. Firmly establishing the causality of a GWAS-nominated regulatory variant requires linking the regulatory variants or the target genes back to the original phenotype. However, due to difficulty for SARS-CoV-2 infection, we cannot assess the expression level of *CCR9* and *SLC6A20* before and after SARS-CoV-2 in peripheral blood mononuclear cells. Further studies in vivo are warranted to investigate the behaviors of immune cells with target gene knockout upon SARS-CoV-2 infection.

## Supplementary information

Supplementary materials

## Data Availability

The online version of this article contains supplementary materials, which is available to authorized users.
