# Supplementary material for: Genome and epigenome editing identify CCR9 and SLC6A20 as target genes at the 3p21.31 locus associated with severe COVID-19
Source: Signal Transduct Target Ther. 2021 Feb 22;6:85. doi: 10.1038/s41392-021-00519-1 (PMC7897877; doi:10.1038/s41392-021-00519-1)
Supplement: Supplementary file 1 — Supplementary materials [file 41392_2021_519_MOESM1_ESM.pdf]

Supplementary Materials for

Genome and epigenome editing identified *CCR9* and *SLC6A20* as target genes at the 3p21.31 locus associated with severe COVID-19

Yao Yao[1,2,†], Fei Ye[3,†], Kailong Li[4,†], Peng Xu[5], Wenjie Tan[3], Quansheng Feng[1], Shuquan Rao[2,†]

1. School of Basic Medicine, Chengdu University of Traditional Chinese Medicine, Chengdu, 611137, China

2. Division of Hematology/Oncology, Boston Children's Hospital, Department of Pediatric Oncology, Dana-Farber Cancer Institute, Harvard Medical School, Boston, 02115, MA, USA

3. NHC Key Laboratory of Biosafety, National Institute for Viral Disease Control & Prevention, Chinese Center for Disease Control and Prevention, China CDC, 102206, Beijing, China

4. Children's Medical Center Research Institute, University of Texas Southwestern Medical Center, Dallas, TX, 75390, USA

5. Hematology Center of Cyrus Tang Medical Institute, Soochow University, Suzhou, 215123, China

[†]These authors contributed equally to this work.

Quansheng Feng and Shuquan Rao are co-corresponding authors.

Correspondence to Shuquan Rao: shuquan.rao@gmail.com

This PDF file includes:

Materials and Methods

Figures. S1 to S5

Tables S1 to S3

Materials and Methods

1. Human cell lines and cell culture

The human bronchial epithelial cell line 16HBE14o- was generously provided by Zhou lab (Channing Division of Network Medicine, Brigham and Women's Hospital, Harvard Medical School). THP-1, HL-60, Jurkat and K562 cells were purchased from ATCC (https://www.atcc.org/).

All the cell lines were incubated at 37°C in a humidified 5% CO2/air incubator. 16HBE14o- cells were maintained in Eagle's Minimal Essential Medium (Gibco, #11095080), supplemented with 10% fetal bovine serum, penicillin (50 units/ml), and streptomycin (50 μg/ml). HL-60, Jurkat and K562 cells were maintained in RPMI 1640 Medium (Gibco, # 11875093) with 10% fetal bovine serum, penicillin (50 units/ml), and streptomycin (50 μg/ml). THP-1 cells were maintained in RPMI 1640 Medium (Gibco, # 11875093) with 10% fetal bovine serum, 2-mercaptoethanol (0.05 mM), penicillin (50 units/ml), and streptomycin (50 μg/ml).

2. Generation of Cas9 and dCas9-expressing stable cell lines

LentiCas9-Blast (Addgene, Cat#52962) and dCas9-KRAB-MeCP2 (Addgene, Cat#110821) were used to transduce cell lines [1]. To produce lentivirus, HEK293T cells were cultured with Dulbecco's Modified Eagle Medium (Gibco, Cat#11995), supplemented with 10% fetal bovine serum (FBS), penicillin/streptomycin (1%) in 15 cm tissue culture dishes. HEK293T cells were transfected at 70–80% confluence in 14 ml of media with 13.3μg pf psPAX2, 6.7μg VSV-G and 20μg of the lentiviral construct plasmid of interest using 180μg of linear polyethylenimine. Then 24 h after transfection, the medium was changed. Lentiviral supernatant was collected at 72 h post-transfection and subsequently concentrated by ultracentrifugation (24,000 rpm for 4 h at 4 °C with a Beckman Coulter SW 32 Ti rotor).

Media was changed after 12 h after lentiviral infection. After 2-d recovery, transduced cells were selected with 10 μg/ml blasticidin for more than 2 weeks. Cas9 activity was confirmed using the pXPR-011 (Addgene, Cat#59702) GFP reporter assay as described previously [2].

3. CRISPR/Cas9 gRNA design, cloning, lentiviral vector production and transduction

CRISPR/Cas9-mediated regional deletion and CRISPRi targeting one potential enhancer within the chromosome 3p21.31 locus were applied in this study.

Single gRNAs used in these experiments were designed with the web tool CRISPOR (http://crispor.tefor.net/). Sequence of all sgRNAs were summarized in Supplementary Table 1. Oligos (from GENEWIZ company) were annealed and ligated into

LentiGuide-Puro (Addgene, Cat#52963). Following lentiviral production and transduction into cell lines with stable SpCas9 expression, 10 μg/ml blasticidin and 1 μg/ml puromycin were added to select for sgRNA expression in cells with stable Cas9 or dCas9 expression.

4. Chromosome immunoprecipitation-qPCR (ChIP)

16HBE14o- cells stably expressing both dCas9-KRAB-MECP2 and specific sgRNAs were crosslinked by incubating with 1% formaldehyde solution (50 mM HEPES–KOH, 100 mM NaCl, 1 mM EDTA, 0.5 mM EGTA, and 1% formaldehyde) for 10 min at room temperature. Glycine was added at a final concentration of 125 mM to stop the reaction. After being washed twice with ice-cold PBS, cells were scraped, pelleted, and lysed in ChIP lysis buffer (10 mM EDTA, 1% SDS, 50 mM pH 8.1 Tris–HCl, and 1% PMSF) for 10 min on ice. The crosslinked chromosome complexes were sonicated to an average fragment size of 200-1000 bp. The ultrasonic products were incubated with anti-Cas9 antibodies (Active Motif, Cat#61757) or IgG isotype control (Cell Signaling Technology, Cat#5415) at 4°C for 1h, and then with protein A/G beads overnight at 4°C. Finally, DNA was dissociated from crosslinked proteins, and purified by DNA purification kit (QIAGEN). Real time qPCR was performed using specific primers (Supplementary table 2).

5. Western blotting

Cells were lysed in 2x Laemmli sample buffer (Bio-Rad, 1610737) and total cellular lysates were then subjected to SDS-PAGE followed by transfer to PVDF membrane. Detection of dCas9-KRAB-MECP2 was done using anti-HA (Cell Signaling Technology, Cat#3724; 1:2000) and HRP-conjugated secondary antibody, followed by Pierce™ ECL Western Blotting Substrate (Thermo Scientific, Cat# 32106). Cofilin (Cell Signaling Technology, Cat#5175; 1:5000) was used as internal control.

6. Clonal expansion of edited cells

Cells after genome editing were seeded for single cell colonies in 96-well plates at a density of 1 cell/well. After 3-week expansion, DNA was purified, and the edited region was amplified by PCR to confirm the deletion of 67.8 Kb genomic region. PCR primers were provided in Supplementary Table 2. PCR products with the edited information were further cloned into pUC57 plasmids for Sanger sequencing.

7. Target gene expression determination by RT-qPCR

Expression of target genes within 1 Mb surrounding the chromosome 3p21.31 locus was confirmed by RT-PCR and RT-qPCR. These genes included *LARS2*, *LIMD1*, *SACM1L*, *SLC6A20*, *LZTFL1*, *CCR9*, *FYCO1*, *CXCR6*, *XCR1*, *CCR1*, *CCR3* and *CCR2*.

RNA was extracted from selected clones using the RNEasy® Plus Mini Kit (Qiagen), and cDNA was synthesized using the iScript™ cDNA Synthesis Kit (Biorad). RT-qPCR was performed with iQ™SYBR® Green Supermix (Biorad) on the CFX96TM Real-Time System (Biorad). Quantification was performed using the ΔΔCT method with β-actin as the reference gene. Gene specific primers were summarized in Supplementary Table 2.

8. Genotyping of SNPs at the 3p21.31 locus in Jurkat cells

A total of 22 SNPs at the 3p21.31 locus were genotyped by Sanger sequencing. Primers covering all the SNPs were listed in Supplementary Table 2.

9. Epigenetic datasets

Epigenetic datasets were obtained from the ENCODE Project (https://www.encodeproject.org/) [1]. Specifically, the following datasets were used in the present study.

10. Statistical analysis

Statistical details including N, mean and statistical significance values are indicated in the text, figure legends, or methods. Error bars represent standard error of the mean (SEM) for each experiment. All statistical analyses were performed using GraphPad Prism, and the detailed information about statistical methods is specified in figure legends or methods. The numbers of independent experiments or biological replicate samples and *P* values (n.s. not significant, **P* < 0.05, ***P* < 0.01, ****P* < 0.001) are provided in individual figures. *P* < 0.05 was considered statistically significant.

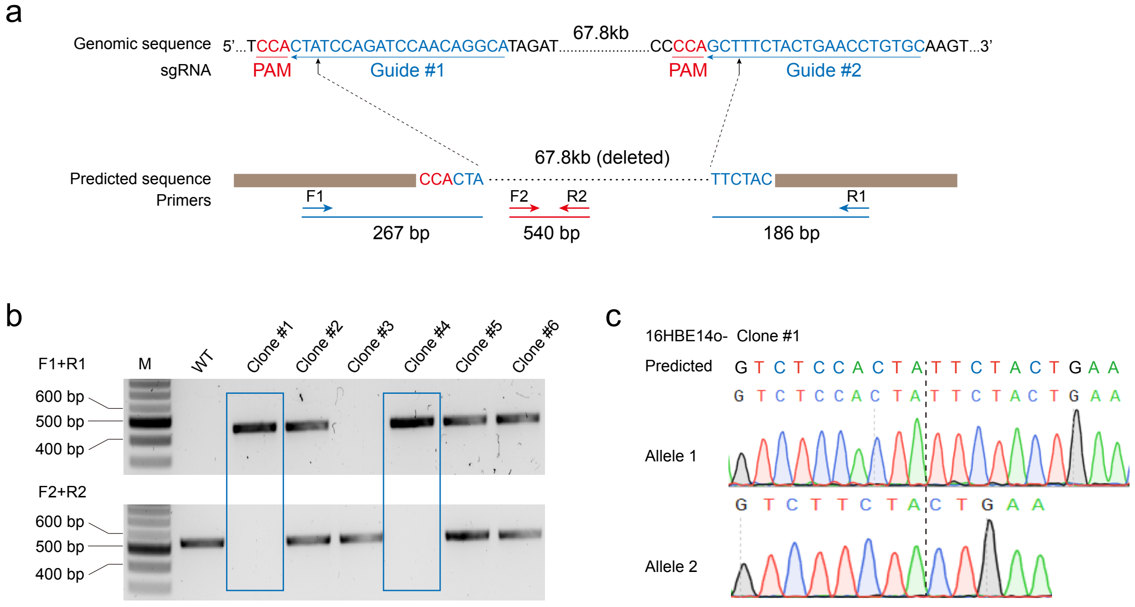
a
Genomic sequence
5'...TCCACTATCCAGATCCAACAGGCATAGAT
67.8kb
CCCCAGCTTTCTACTGAACCTGTGCAAGT...3'
sgRNA
PAM
Guide #1
PAM
Guide #2
67.8kb (deleted)
Predicted sequence
CCACTA
TTCTAC
Primers
F1
F2
R2
R1
267 bp
540 bp
186 bp
b
F1+R1
M
WT
Clone #1
Clone #2
Clone #3
Clone #4
Clone #5
Clone #6
600 bp
500 bp
400 bp
F2+R2
600 bp
500 bp
400 bp
c
16HBE14o- Clone #1
Predicted
G T C T C C A C T A T T C T A C T G A A
Allele 1
G T C T C C A C T A T T C T A C T G A A
Allele 2
G T C T T C T A C T G A A

Figure. S1.

Selection of isogenic clones with 67.8 Kb deletion the chromosome 3p21.31 locus in 16HBE14o- cells

(a) Schematic diagram of genome editing strategy. The positions of sgRNAs and PAM sequences are shown in blue and red, respectively. The locations of PCR primers are indicated by arrows. F, forward; R, reverse.

(b) Clones of bi-allelic deletion are indicated by presence of PCR product from F1 + R1 but absence of PCR product from F2 + R2.

(c) Examples of Sanger sequencing showing genomic seqeunces after bi-allelic 67.8 Kb deletion.

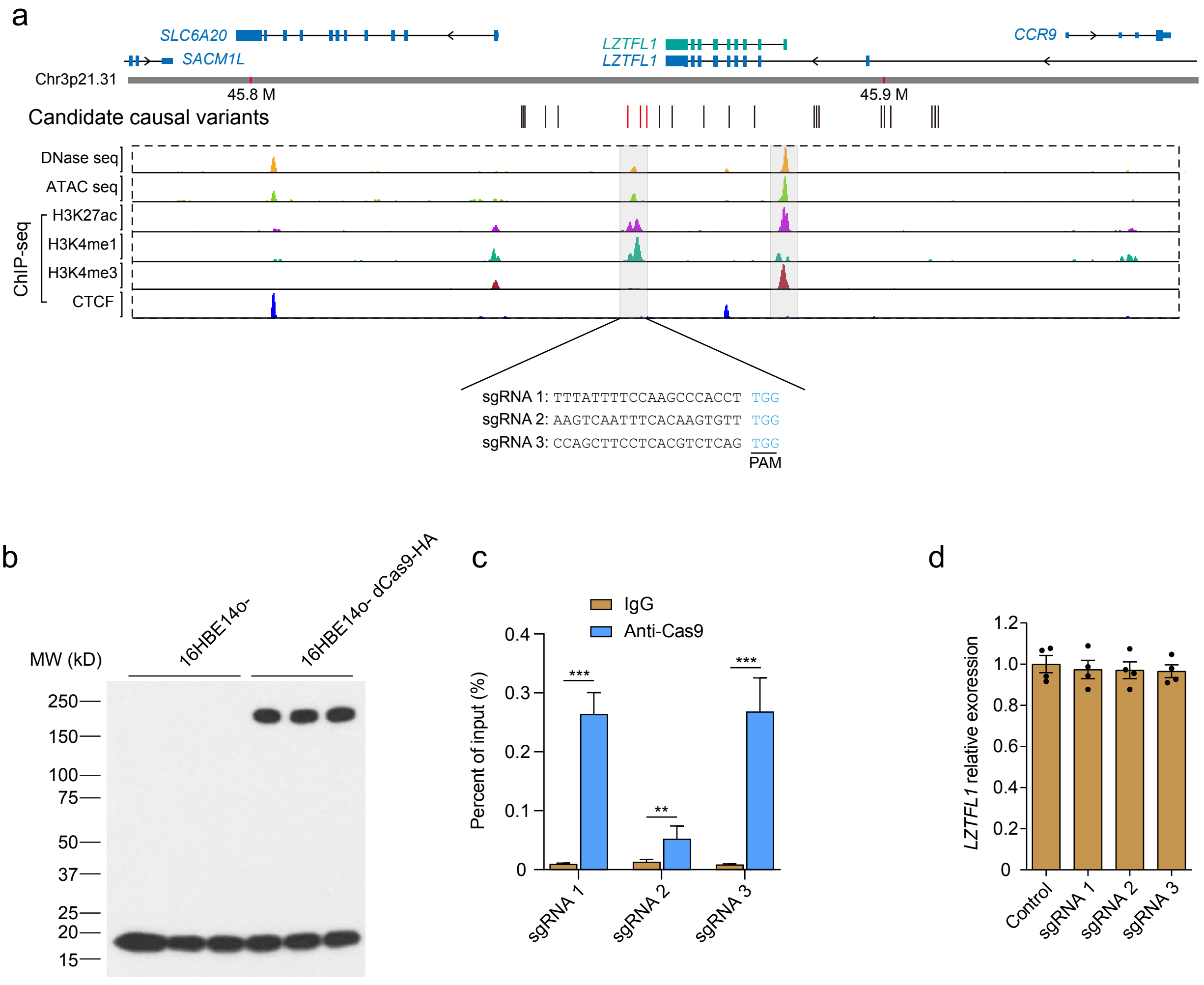

a
SLC6A20
SACM1L
LZTFL1
LZTFL1
CCR9
Chr3p21.31
45.8 M
45.9 M
Candidate causal variants
DNase seq
ATAC seq
H3K27ac
H3K4me1
H3K4me3
CTCF
ChIP-seq
sgRNA 1: TTTATTTTCCAAGCCCACCT TGG
sgRNA 2: AAGTCAATTTCACAAGTGTT TGG
sgRNA 3: CCAGCTTCCTCACGTCTCAG TGG
PAM
b
MW (kD)
16HBE14o-
16HBE14o- dCas9-HA
250
150
100
75
50
37
25
20
15
c
IgG
Anti-Cas9
Percent of input (%)
0.4
0.3
0.2
0.1
0
***
**
***
sgRNA 1
sgRNA 2
sgRNA 3
d
LZTFL1 relative exoression
1.2
1.0
0.8
0.6
0.4
0.2
0
Control
sgRNA 1
sgRNA 2
sgRNA 3

Figure. 2.

Epigenetic signature and CRISPRi at the chromosome 3p21.31 locus in human lung bronchial epithelium

(a) Epigenetic signature at the chromosome 3p21.31 locus in A549 cells. Density maps are shown for DNase-Seq and ATAC-Seq, ChIP-seq of active histone marks (H3K27ac and H3K4me1), promoter mark (H3K4me3) and CTCF. CRISPRi was performed with sgRNAs targeting one potential enhancere region. Locations of candiate causal variants from fine-mapping analysis were indicated by short vertiical lines (both in black and in red). Genomic locations in hg19 was presented.

(b) Western blotting analysis indicated expression of dCas9-KRAB-MECP2 in transduced 16HBE14o- cells. Cofilin was used as internal control (n = 3 biological replicates).

(c) ChIP-qPCR of sgRNA binding to the target locus. Primers specific for the sgRNA binding sites were used for the qPCR (n = 3 biological replicates).

an increase in the proliferation marker SOX2 in TCF20 depleted NSCs

(d) *LZTFL1* expression was determined by real time qPCR (n = 3 technical replicates). Statistics: student's t test; ****P* < 0.001, ***P* < 0.01.

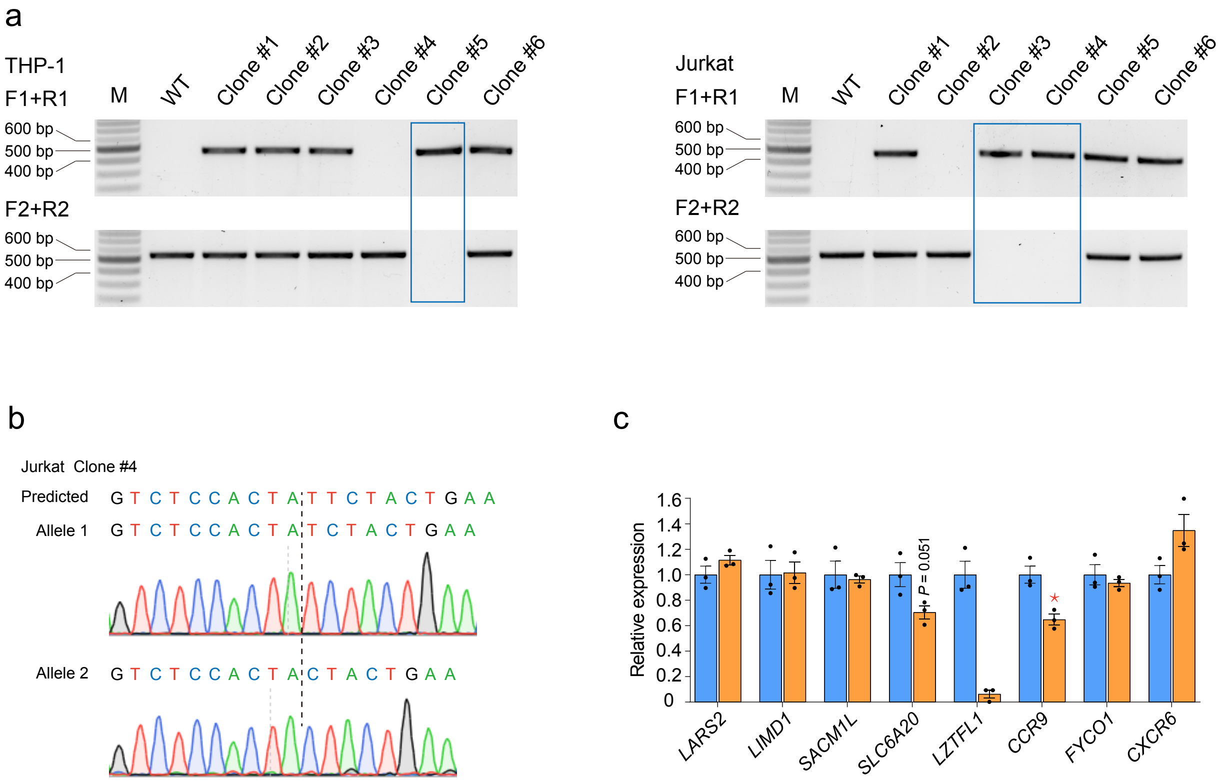
a
THP-1
F1+R1
M
WT
Clone #1
Clone #2
Clone #3
Clone #4
Clone #5
Clone #6
600 bp
500 bp
400 bp
F2+R2
Jurkat
b
Jurkat Clone #4
Predicted G T C T C C A C T A T T C T A C T G A A
Allele 1 G T C T C C A C T A T C T A C T G A A
Allele 2 G T C T C C A C T A C T A C T G A A
c
Relative expression
1.6
1.4
1.2
1.0
0.8
0.6
0.4
0.2
0
P = 0.051
LARS2
LIMD1
SACM1L
SLC6A20
LZTFL1
CCR9
FYCO1
CXCR6

Figure. 3.

Selection of isogenic clones with 67.8 Kb deletion the chromosome 3p21.31 locus in THP-1 and Jurkat cells

Clones of bi-allelic deletion are indicated by presence of PCR product from F1+R1 but absence of PCR product from F2+R2.

a

RSID	Posterior probability	Genotype in Jurkat
rs13071258	0.015	GG
rs17763537	0.018	CC
rs17763569	0.022	GG
rs17763742	0.014	AA
rs17712877	0.016	GG
rs17713054	0.040	GG
rs13078854	0.049	GG
rs71325088	0.049	TT
rs10490770	0.052	TT
rs35624553	0.043	AA
rs67959919	0.055	GG
rs11385942	0.114	AA
rs35508621	0.061	TT
rs34288077	0.046	AA
rs35081325	0.057	AA
rs35731912	0.072	CC
rs34326463	0.042	AA
rs76374459	0.024	GG
rs73064425	0.049	CC
rs13081482	0.040	AA
rs35652899	0.055	CC
rs35044562	0.032	AA

b

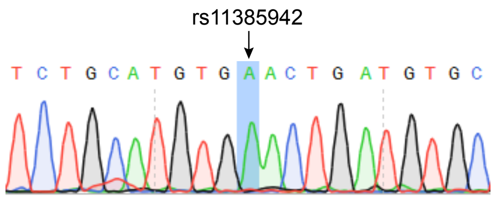

Figure. 4.

Genotypes of 22 candidate causal SNPs at the 3p21.31 locus

(a) Summary of genotypes of 22 candidate SNPs at the 3p21.31 locus. Posterior probability of each SNP is derived from Bayesian fine-mapping analysis (see the original manuscript) [2].

(b) Example of sequencing chromatograms (rs11385942 in Jurkat cells).

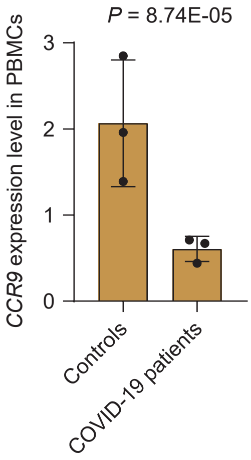

P = 8.74E-05
CCR9 expression level in PBMCs
3
2
1
0
Controls
COVID-19 patients

Figure. 5.

Decreased expression of *CCR9* in PBMCs from COVID-19 patients

RNA-Seq datasets were publicly available [3]. PBMCs were isolated from peripheral blood from both COVID-19 patients (n =3) and healthy controls (n = 3). Gene expression was calculated by featureCounts in SubReads package (v1.5.3) with the "-M" parameter [4]. Differentially expressed genes were called by using DESeq2 package (v1.26.0) [5].

PBMC, peripheral blood mononuclear cells.

Table S1. Sequence of sRNAs used in the present study

**For genomic deletion**	
Guide #1 (forward)	TGCCTGTTGGATCTGGATAG
Guide #2 (reverse)	GCACAGGTTCAGTAGAAAGC

**For CRISPRi**	
Guide # 1	TTTATTTTCCAAGCCCACCT
Guide # 2	AAGTCAATTTCACAAGTGTT
Guide # 3	CCAGCTTCCTCACGTCTCAG

Table S2. Sequence of primers used in the present study

For identification of genomic deletion clones

Oligos	Sequence	Product size
Primer F1	CCTCCTGAGTAGCTGAGATTA	453 bp
Primer R1	CTAGAACCAGAACTTGGCTAAA	
Primer F2	TCACTCAGGACAGACTTACC	540 bp
Primer R2	GACCAGCATGGACAACATAG	

For real time qPCR

Oligos	Sequence	Product size
LARS2_F	TGGATGCCTGTGGATTTG	202 bp
LARS2_R	CCACTTCCTCTCTCTGTAGAT	
LIMD1_F	TCACCCGAAGGCTGATTA	109 bp
LIMD1_R	GAAGCATGTGTCATGGTAGAG	
SLC6A20_F	TGCCTACCCAAGCAAATAC	116 bp
SLC6A20_R	CCTCTGTGTAGACGATGAATG	
SACM1L_F	CATTGCCTTTGACTTCCATAAG	197 bp
SACM1L_R	TCTGGATCACATTGGTTCTATC	
CCR9_F	CCCTGATACAAGCCAAGAAG	143 bp
CCR9_R	GGAGATGAACATGGCATAGG	
LZTFL1_F	CACTGTCGCTGCCTTAAA	118 bp
LZTFL1_R	CTCCTGAACCCTGAGTAGAT	
FYCO1_F	GATGAGGAATTGTGCCAGATA	117 bp
FYCO1_R	CGTTAGCGAGGTTGATGTAG	
CXCR6_F	CAGCACACACTGGGAATAC	105 bp
CXCR6_R	GCATAGAGCACAGGGTTAAG	
XCR1_F	TTCTGCAGACGCTGTTTC	126 bp
XCR1_R	CATAGAGCACCGGGTTAAAG	
CCR1_F	CCTCTGTACTCCTTGGTATTTG	103 bp
CCR1_R	GGTAGATGCTGGTCATGTTT	
CCR3_F	CACCCTACAATGTGGCTATC	168 bp
CCR3_R	GGAACCTCTCTCCAACAAAG	
CCR2_F	CTCGCTGGTGTTCATCTTT	149 bp
CCR2_R	CCCACAATGGGAGAGTAATAAG	

For ChIP-qPCR

Guide_#1 F	CATCCTGAAGCTACGTACAA	137 bp
Guide_#1 R	GGTTTATGCGTGTTCTTTCC	
Guide_#2 F	TACTTGGCCAGGAAACAG	83 bp
Guide_#2 R	GCAGAGCCCGTCATTAT	
Guide_#3 F	ATTAAGGCCTGGTGGATAC	122 bp
Guide_#3 R	GAGGTAGGAAAGGGAAGAAA	

For genotyping of SNPs at chr 3p21.31

Oligos	Sequence	Product size
rs13071258_1F	TCCTGAGTAGCTGGGATTA	524 bp
rs13071258_1R	ATTTGTACTGTGTGAGTCTTTG	
rs17763742_1F	CTTGGCCAGTCTATTGTATTTC	368 bp
rs17763742_1R	CCTGTCTTACTTTGTGCTTTAT	
rs17712877_1F	AGTTGGAGGCTGTAATGAG	372 bp
rs17712877_1R	CTGTGTCCCATTAGTTGTTATC	
rs17713054_1F	TGCAGTGCAGGACAATAA	547 bp
rs17713054_1R	CATCCCACAGTGAACTAAGA	
rs13078854_1F	CGTAAGGCCAGAAGAACA	434 bp
rs13078854_1R	CTGTGGTGGTGCTTTAGA	
rs71325088_1F	GTCAGAGGTCCAACAATCT	314 bp
rs71325088_1R	CCCTGTCCTGCTGTATTT	
rs10490770_1F	GAGTTTAGTCTGCCGTATCA	519 bp
rs10490770_1R	TGTCATCAGCTCCTTCATC	
rs35624553_1F	GCAGGATGGATTTGACTTATC	517 bp
rs35624553_1R	ATCCCACACACCTCTCTA	
rs67959919_2F	CAATGAGAGTATGACCACTAGA	429 bp
rs67959919_2R	CTTCCTGGTATGCAAGATATTC	
rs11385942_1F	CACCACCTTCTCAGAGTTT	452 bp
rs11385942_1R	TGGTCAGTATTACCATCAGTC	

Oligos	Sequence	Product size
rs35508621_1F	GTCTTTAAGGTCTGGAGGATAG	563 bp
rs35508621_1R	GTGGCTCACCTCTGTAATC	
rs34288077_1F	GTTTCAGTGCCTGCATAATAG	461 bp
rs34288077_1R	CAATGCTGTGGAGGAGAA	
rs35081325_1F	GCAGCAGCTTGAGTAGAT	498 bp
rs35081325_1R	CATTCACAGCAGAGAGAAATG	
rs34326463_1F	GCCTCCCACTCCTTATTATC	533 bp
rs34326463_1R	CCCATTCTGGCTACAGTATT	
rs76374459_1F	TGTCCAAGAAATAGGGACATAG	347 bp
rs76374459_1R	TTGAGGCCAGGAGTTAGA	
rs73064425_2F	CCATGGTGCCTGACTATTT	405 bp
rs73064425_2R	CCTGTAATCCCAGCTACTTG	
rs13081482_1F	CACCATACAGTCAGCTCTAC	459 bp
rs13081482_1R	CCCAAAGTGCTGGGATTA	
rs35652899_1F	GAGGCTATCCCACAACAA	502 bp
rs35652899_1R	TGCCCATCATTACAAGTTTAC	
rs35044562_1F	GGCAGAGAGTAAACTTGTAATG	535 bp
rs35044562_1R	CTTGTTCGTTGTCGTTGTT	

Table S3. Summary of epigenetic data sources from the ENCODE and ROADMAP projects used in the present study

No.	cell type	Assay	xperiment accession i	GEO Acc. IDs	Source
1	human primary T cell	H3K27ac ChIP-seq	ENCSR222QLW	GSM1058764	ROADMAP
2	human primary T cell	H3K4me1 ChIP-seq	ENCSR218OEZ	GSM1058778	ROADMAP
3	human primary T cell	H3K4me3 ChIP-seq	ENCSR395YXN	GSM1058782	ROADMAP
4	human primary T cell	H3K9me3 ChIP-seq	ENCSR115GFR	GSM1058783	ROADMAP
5	human primary T cell	DNase-seq	ENCSR627UDJ	GSM774201	ROADMAP
6	human primary B cell	H3K27ac ChIP-seq	ENCSR191ZQT	GSM1027287	ENCODE
7	human primary B cell	H3K4me1 ChIP-seq	ENCSR290YLQ	GSE96030	ENCODE
8	human primary B cell	H3K4me3 ChIP-seq	ENCSR878JSF	GSE101354	ENCODE
9	human primary B cell	H3K9me3 ChIP-seq	ENCSR005WWZ	GSE100949	ENCODE
10	human primary B cell	DNase-seq	ENCSR891VOV	GSM701493	ROADMAP
11	human CD14-positive monocyte	H3K27ac ChIP-seq	ENCSR000ASJ	GSM1003559	ENCODE
12	human CD14-positive monocyte	H3K4me1 ChIP-seq	ENCSR000ASM	GSM1003535	ENCODE
13	human CD14-positive monocyte	H3K4me3 ChIP-seq	ENCSR000ASN	GSM1003536	ENCODE
14	human CD14-positive monocyte	H3K9me3 ChIP-seq	ENCSR000ASP	GSM1003538	ENCODE
15	human CD14-positive monocyte	DNase-seq	ENCSR695AUY	GSM701503	ROADMAP
16	K562 cell line	H3K27ac ChIP-seq	ENCSR000AKP	GSM733656	ENCODE
17	K562 cell line	H3K4me1 ChIP-seq	ENCSR000EWC	GEO:GSM788085	ENCODE
18	K562 cell line	H3K4me3 ChIP-seq	ENCSR668LDD	GEO:GSE96303	ENCODE
19	K562 cell line	H3K9me3 ChIP-seq	ENCSR000APE	GEO:GSM733776	ENCODE
20	K562 cell line	DNase-seq	ENCSR000EOT	eEH003153;UCSC-ENC	ENCODE
21	A549	ATAC-seq	ENCSR220ASC	GSE114202	ENCODE
22	A549	H3K27ac ChIP-seq	ENCSR410BCN	GSE91258	ENCODE
23	A549	H3K4me1 ChIP-seq	ENCSR636PIN	GSE91306	ENCODE
24	A549	H3K4me3 ChIP-seq	ENCSR203XPU	GSE91218	ENCODE
25	A549	CTCF ChIP-seq	ENCSR035OXA	GSE92782	ENCODE
26	A549	DNase-seq	ENCSR136DNA	GSE91208	ENCODE

References

1 Consortium, E. P. An integrated encyclopedia of DNA elements in the human genome. *Nature* **489**, 57-74 (2012).

2 Severe Covid, G. G. *et al.* Genomewide Association Study of Severe Covid-19 with Respiratory Failure. *N Engl J Med* **383**, 1522-1534 (2020).

3 Xiong, Y. *et al.* Transcriptomic characteristics of bronchoalveolar lavage fluid and peripheral blood mononuclear cells in COVID-19 patients. *Emerg Microbes Infect* **9**, 761-770 (2020).

4 Liao, Y., Smyth, G. K. & Shi, W. featureCounts: an efficient general purpose program for assigning sequence reads to genomic features. *Bioinformatics* **30**, 923-930 (2014).

5 Love, M. I., Huber, W. & Anders, S. Moderated estimation of fold change and dispersion for RNA-seq data with DESeq2. *Genome Biol* **15**, 550 (2014).
